# Strategies to improve the quality of life of persons post-stroke: protocol of a systematic review

**DOI:** 10.1186/s13643-017-0579-3

**Published:** 2017-09-07

**Authors:** Sarah E.P. Munce, Laure Perrier, Saeha Shin, Chamila Adhihetty, Kristen Pitzul, Michelle L.A. Nelson, Mark T. Bayley

**Affiliations:** 1 Brain and Spinal Cord Rehabilitation Program, Toronto Rehabilitation Institute-University Health Network, University of Toronto, 550 University Avenue, Toronto, Ontario M5G 2A2 Canada; 2 University of Toronto, Gerstein Science Information Centre, 9 King’s College Circle, Toronto, Ontario M5S 1A5 Canada; 3 Dalla Lana School of Public Health, University of Toronto, 27 King’s College Circle, Toronto, Ontario M5S 1A1 Canada; 4 Institute of Health Policy, Management and Evaluation, University of Toronto, 4th floor, 155 College Street, Toronto, Ontario M5T 3M6 Canada; 5 Lunenfeld-Tanenbaum Research Institute; Sinai Health System, 1 Bridgepoint Drive, Toronto, Ontario M4M 2B5 Canada

**Keywords:** Stroke, Quality improvement, Knowledge translation, Quality of life, Systematic review, Protocol

## Abstract

**Background:**

While many outcomes post-stroke (e.g., depression) have been previously investigated, there is no complete data on the impact of a variety of quality improvement strategies on the quality of life and physical and psychological well-being of individuals post-stroke. The current paper outlines a systematic review protocol on the impact of quality improvement strategies on quality of life as well as physical and psychological well-being of individuals with stroke.

**Methods:**

MEDLINE, CINAHL, EMBASE, and PsycINFO databases will be searched. Two independent reviewers will conduct all levels of screening, data abstraction, and quality appraisal. Only randomized controlled trials that report on the impact of quality improvement strategies on quality of life outcomes in people with stroke will be included. The secondary outcomes will be physical and psychological well-being. Quality improvement strategies include audit and feedback, case management, team changes, electronic patient registries, clinician education, clinical reminders, facilitated relay of clinical information to clinicians, patient education, (promotion of) self-management, patient reminder systems, and continuous quality improvement. Studies published since 2000 will be included to increase the relevancy of findings. Results will be grouped according to the target group of the varying quality improvement strategies (i.e., health system, health care professionals, or patients) and/or by any other noteworthy grouping variables, such as etiology of stroke or by sex.

**Discussion:**

This systematic review will identify those quality improvement strategies aimed at the health system, health care professionals, and patients that impact the quality of life of individuals with stroke. Improving awareness and utilization of such strategies may enhance uptake of stroke best practices and reduce inappropriate health care utilization costs.

**Systematic review registration:**

PROSPERO, CRD42017064141

**Electronic supplementary material:**

The online version of this article (10.1186/s13643-017-0579-3) contains supplementary material, which is available to authorized users.

## Background

Stroke is a major cause of death, loss of independence, and decreased quality of life [1–3]. Although care for individuals with stroke has previously focused on the acute phase, there is a significant group of patients who have persistent disabilities many years post-stroke [4, 5]. These disabilities can include physical limitations, such as paralysis or fatigue [6–8], and/or cognitive and psychological issues, such as depression and/or anxiety [9, 10]. While many of these outcomes (e.g., depression) have been investigated in great depth, there is no complete data to date, on the impact of a variety of quality improvement strategies on the quality of life and physical and psychological well-being of individuals post-stroke. Quality improvement is defined as the combined efforts of healthcare professionals, patients and their families, researchers, payers, planners, and educators to make changes that will lead to better system performance (care), professional development (learning), and patient outcomes (health) [11]. This paper outlines the protocol for a systematic review on the impact of quality improvement strategies on the quality of life and physical and psychological well-being of individuals with stroke.

## Methods/design

This protocol is informed by the guidelines from the Cochrane Collaboration [12], and the final report will conform to the Preferred Reporting Items for Systematic Reviews and Meta-Analyses (PRISMA) [13]. The current protocol conforms to the PRISMA-Protocols (PRISMA-P) checklist and has been included as an Additional file 1. This protocol was registered with the PROSPERO database (CRD42017064141).

### Eligibility criteria

Our systematic review will include randomized controlled trials only, including cluster randomized controlled trials. English language trials published in the last 17 years (i.e., since 2000) will be included. This time frame was selected to ensure the relevancy of the findings in the current healthcare context as well as feasibility. Trials will be eligible if they examine a predefined list of quality improvement strategies for adults (≥ 18 years of age) post-stroke. Quality improvement strategies will include those targeted at health systems (e.g., team changes), health care professionals (e.g., professional reminders), or patients (e.g., reminders). The particular strategies will include audit and feedback, case management, team changes, electronic patient registries, clinician education, clinical reminders, facilitated relay of clinical information to clinicians, patient education, (promotion of) self-management, patient reminder systems, and continuous quality improvement. This list of quality improvement strategies has been previously identified through other systematic reviews and meta-analyses (i.e., Shojania et al., 2006; Tricco et al., 2012) [14, 15]. Outcomes of interest will be quality of life (primary) and physical and psychological well-being (secondary). Quality of life is operationalized as an individual’s perception of position in life in the context of the culture and value systems in which he or she lives and in relation to their goals, expectations, standards, and concerns [16]. Well-being is defined as (1) a subjective or objective perception of improvement in physical health or of symptoms related to stroke or to side effects of treatment, and/or (2) a subjective or objective perception of improvement of psychological functioning [16]. Quality of life must be specifically identified as the primary outcome measure to be included in the analysis. Furthermore, studies must report on quality of life as measured by validated scales, classifications, and measurement systems (e.g., the Short-Form-36 (SF-36), the Individual Quality of Life Interview). These measures must have been previously used in a stroke population. Similarly, studies will only be included if they report on well-being as measured by validated and specific standardized impairment, distress, or psychological scales (e.g., Center for Epidemiologic Studies Depression Scale (CESD) [17], Hospital Anxiety and Depression Scale (HADS) [18]), and if they have been previously used in a stroke population.

### Information sources and literature search

Literature search strategies will be developed using medical subject headings (MeSH) and text words related to quality improvement strategies in post-stroke management. Studies will be identified by searching MEDLINE (OVID interface, 2000 onwards), CINAHL (EBSCO interface, 2000 onwards), EMBASE (OVID, 2000 to present), and PsycINFO (OVID interface, 2000 onwards). The search strategy for MEDLINE can be found in Additional file 2. In addition to the electronic databases, grey literature (i.e., unpublished and difficult to locate material) will be searched. Unpublished material will be identified by searching the Dissertations and Theses database as well as searching for relevant abstracts from conference proceedings via the Conference Papers Index (e.g., Canadian Stroke Congress conference). Finally, experts in the field of stroke and implementation science (including one of the authors, MB) will be contacted and consulted in order to ensure that all relevant data is obtained. An experienced information specialist (LP) will conduct all of the literature searches.

### Study selection process

To increase reliability of screening among reviewers, a pilot-test of a pre-defined screening form based on the eligibility criteria outlined above (i.e., the “Eligibility criteria” section) will be performed on a random 1% sample. The inclusion and exclusion criteria will be discussed and clarified to promote the consistent application of the selection criteria (e.g., reviewers are aware of what constitutes a quality improvement strategy), if necessary. Two reviewers will independently screen the titles and abstracts identified by the literature search for inclusion using the screening form (i.e., level 1 screening). The full text of the potentially relevant articles will then be acquired and screened to determine final inclusion (i.e., level 2 screening). Both reviewers must also determine that an outcome measure in the stated domains of interest have adequate psychometrics. Discussion or the involvement of a third reviewer will be available to resolve discrepancies. Studies excluded during the screening phase will be documented along with an explanation for exclusion.

### Data items and data collection process

Data to be abstracted from the publications will include study characteristics (e.g., author names, year of publication, country of study conduct, study design, sample size), participant characteristics (e.g., etiology of stroke (i.e., ischemic or hemorrhagic), mean age and standard deviation, stroke latency, residential status, stroke severity, etc.), quality improvement characteristics (e.g., type and number of quality improvement strategies), and outcome results (e.g., specific scale/measure of quality of life, specific scale/measure of well-being, such as depressive symptoms, social support, and physical health symptoms). We will make note of trials that included quality of life as an outcome but did not include or specify it as a primary outcome.

As in the study selection process, a data abstraction form will be pilot tested, standardized, and modified if poor agreement is observed. For example, wording on the form that may be contributing to poor agreement will be reviewed and modified. Two reviewers will independently abstract all of the data and discussion, or a third reviewer will resolve discrepancies. DistillerSR will be used to manage level 1 and 2 screening as well as data abstraction and quality appraisal.

### Methodological quality/risk of bias appraisal

A standardized quality assessment tool for randomized controlled trials, the EPOC Risk of Bias Tool [12], will be applied to appraise the methodological quality and risk of bias of the included studies.

### Synthesis of included studies

The results of the systematic review will be summarized descriptively. Sub-group analysis will likely be conducted by quality improvement strategy type and/or by any other noteworthy grouping variable (e.g., by etiology of stroke, sex, time points for outcomes). If low statistical (e.g., *I* ^2^ < 60%) [19], methodological, and clinical heterogeneity is observed, random effects meta-analysis will be performed [20]. The mean difference will be used for continuous outcomes (e.g., the SF-36 quality of life scale), and the relative risk will be used for dichotomous outcomes (e.g., Composite International Diagnostic Interview Short Form (CIDI-SF) [21]; depression (yes/no)). Since many issues suitable for sensitivity analysis are only identified during the review process, as stated by the Cochrane Handbook [22], we will do a thorough examination of the results to determine if this is necessary. All analyses will be conducted in Review Manager Version 5.3 (available at http://community.cochrane.org/tools/review-production-tools/revman-5).

## Discussion

This protocol will lead to the first systematic review of the impact of quality improvement strategies on quality of life after stroke. At the end of the review, a robust end-of-project knowledge translation strategy will be implemented. The results of the systematic review will be presented at relevant meetings both locally/nationally (e.g., Canadian Stroke Congress conference) and internationally (e.g., European Stroke Conference) and published in an open access peer-reviewed journal so that results are accessible to the appropriate scientific and clinical audiences. The findings will also be disseminated through the newsletters (print and on-line) of interested organizations, such as the Heart and Stroke Foundation of Canada. Lastly, linkages with local clinical programs (e.g., rehabilitation programs) and/or research initiatives will be made for timely and effective application of the research findings.

This systematic review will identify those quality improvement strategies aimed at the health system, health care professionals, and patients that impact the quality of life and physical and psychological well-being of individuals with stroke. Knowledge and application of such quality improvement strategies may reduce inappropriate health care utilization costs, such as acute care inpatient readmission.

## Additional files

Additional file 1: PRISMA-P 2015 Checklist. (DOCX 36 kb)

Additional file 2: Search strategy for MEDLINE. (DOC 29 kb)

## Abbreviations

CESD: Center for Epidemiologic Studies Depression Scale
CIDI-SF: Composite International Diagnostic Interview Short Form
HADS: Hospital Anxiety and Depression Scale
PRISMA: Preferred Reporting Items for Systematic Reviews and Meta-Analyses
PRISMA-P: Preferred Reporting Items for Systematic Reviews and Meta-Analyses Protocols
SF-36: Short-Form-36
