# Supplementary material for: Strategies to improve the quality of life of persons post-stroke: protocol of a systematic review
Source: Syst Rev. 2017 Sep 7;6:184. doi: 10.1186/s13643-017-0579-3 (PMC5590115; doi:10.1186/s13643-017-0579-3)
Supplement: Supplementary file 2 — Search strategy for MEDLINE. (DOC 29 kb) [file 13643_2017_579_MOESM2_ESM.doc]

Database: Ovid MEDLINE: Epub Ahead of Print, In-Process & Other Non-Indexed Citations, Ovid MEDLINE® Daily and Ovid MEDLINE® <1946-Present>

Search Strategy:

--------------------------------------------------------------------------------

1. Case Management/ [ Quality Improvement ]
2. Disease Management/
3. "Delivery of Health Care, Integrated"/
4. Progressive Patient Care/
5. "Patient-Centered Care"/
6. "Patient Care Planning"/
7. (case adj management).tw.
8. (care adj management).tw.
9. (disease adj management).tw.
10. ("patient centred" adj care).tw.
11. ("patient centered" adj care).tw.
12. Medical Records Systems, Computerized/
13. Drug Therapy, Computer-Assisted/
14. Clinical Pharmacy Information Systems/
15. Telemedicine/
16. Decision Support Systems, Clinical/
17. Decision-Making Computer Assisted/
18. "computer$ decision making".tw.
19. (decision$ adj2 support$).tw.
20. (decision$ adj2 tool$).tw.
21. telemedicine.tw.
22. interprofession$.tw.
23. inter-profession$.tw.
24. interdisciplin$.tw.
25. inter-disciplin$.tw.
26. Interprofessional Relations/
27. multiprofession$.tw.
28. multi-profession$.tw.
29. multidisciplin$.tw.
30. multi-disciplin$.tw.
31. Self Care/
32. self-manag$.tw.
33. (self adj manag$).tw.
34. self-car$.tw.
35. (self adj car$).tw.
36. self-monitor$.tw.
37. (self adj monitor$).tw.
38. Reminder Systems/
39. "Appointments and Schedules"/
40. remind$.tw.
41. exp Reimbursement, Incentive/
42. Physician Incentive Plans/
43. incentive$.tw.
44. (pay$ adj2 perform$).tw.
45. (audit$ adj2 feedback).tw.
46. Clinical Audit/
47. Medical Audit/
48. Nursing Audit/
49. Total Quality Management/
50. CQI.tw.
51. TQM.tw.
52. "total quality management".tw.
53. "continuous quality improvement".tw.
54. Patient Education as Topic/
55. Education, Continuing/
56. Education, Medical, Continuing/
57. Education, Nursing, Continuing/
58. (professional adj development).tw.
59. educat$.tw.
60. or/1-59
61. exp Cerebrovascular Disorders/ [ Stroke ]
62. Stroke/
63. stroke.tw.
64. poststroke.tw.
65. post-stroke.tw.
66. cerebrovasc$.tw.
67. (cerebral adj vasc$).tw.
68. Hemiplegia/
69. hemipleg$.tw.
70. hemipar$.tw.
71. or/61-70
72. adult.mp. [ Adult filter – validated, highly sensitive ]
73. Middle Aged/
74. age$.tw.
75. exp Adult/
76. or/72-75
77. randomized controlled trial.pt. [ RCT fitler - validated ]
78. randomized.mp.
79. placebo.mp.
80. or/77-79
81. 60 and 71 and 76 and 80
82. exp Animals/ not (exp Animals/ and Humans/)
83. 81 not 82
84. limit 83 to (english language and yr="2000 -Current")
